# Correlation between ultrasonography and elastography parameters and molecular subtypes of breast cancer in young women

**DOI:** 10.1080/07853890.2024.2443041

**Published:** 2024-12-28

**Authors:** Dian-xia Men, Hui-zhan Li, Juan Dong, Meng-hua Xue, Zhi-fen Wang, Wen-li Xiao, Ji-ping Xue, Mei-hong Jia

**Affiliations:** Department of Ultrasonographl, Shanxi Bethune Hospital, Shanxi Academy of Medical Sciences, Third Hospital of Shanxi Medical University, Tongji Shanxi Hospital, Taiyuan, Shanxi Province, China

**Keywords:** Breast cancer, molecular subtype, shear wave elastography, ultrasonography, young women

## Abstract

**Objective:**

To explore the differences of conventional ultrasound characteristics, elastic imaging parameters and clinicopathological characteristics of distinct molecular subtypes of breast cancer in young women, and to identify imaging parameters that exhibited significant associations with each molecular subtype.

**Methods:**

We performed a retrospective analysis encompassing 310 young women with breast cancer. Observations were made regarding the ultrasonography and elastography characteristics of the identified breast lesions. Subsequently, based on immunohistochemistry results patients were classified into five distinct molecular subtypes: luminal A, luminal B (HER2−), luminal B (HER2+), HER2+, and triple-negative breast cancer (TNBC). Clinical, pathological, and ultrasound imaging features were compared among these subtypes using binary logistic regression analysis.

**Results:**

Statistically significant differences were observed in various parameters across the five molecular subtypes (*p* < 0.05), including tumor size, morphology, margins, calcification, posterior echo features, blood flow (Adler grading), and tumor hardness. Specifically, luminal A subtype exhibited propensity for spiculated margins, lower blood flow grading, and decreased hardness; luminal B subtype was characterized by angular margins; HER2+ subtype manifested higher blood flow grading, calcification, and elevated hardness. Conversely, TNBC subtype displayed smooth margins, absence of calcification, and heightened hardness.

**Conclusion:**

Specific molecular subtypes of breast cancer have unique ultrasonic and elastic imaging characteristics.

## 1. Introduction

In 2020, breast cancer emerged as the most prevalent cancer globally and the foremost newly diagnosed cancer among Chinese women [1]. In China, although the data of 66,201 registered breast cancer patients from 2007 to 2020 show that breast cancer patients under 40 years old account for less than 15% [2], it is worth noting that the proportion of newly diagnosed breast cancer patients under 40 years old is increasing year by year, from 11.4% in 1999 to 16.4% in 2017 [3], and the ratio of breast cancer patients aged ≤ 40 increased from 17.7% in 2015 to 22.3% in 2021 [4]. Moreover, these patients encounter unique challenges compared to older counterparts, including concerns regarding body image, employment, fertility, and childcare [5]. Young patients with breast cancer have more opportunities and needs to participate in the workplace after being cured. They assume more complex social and family roles, and have higher requirements for quality of life. During the anti-tumor treatment, young breast cancer patients have personalized needs such as fertility protection and breast shape retention [4]. Therefore, management of young breast cancer patients is facing great challenges and needs to pay more attention in clinical practice.

Compared with older patients, young breast cancer patients have a higher risk of recurrence and metastasis and a poor overall survival. This difference may be related to the aggressive biological behaviour of young breast cancer. When compared with older patients, young women with breast cancer had higher-grade tumors, more probability of lymphovascular invasion in tumor, and more triple-negative subtype [6]. Given the heterogeneous nature of breast cancer, which encompasses diverse morphological and biological attributes, along with multiple immunohistochemical subtypes, substantial variations exist in clinical presentations, treatment responses, and prognostic outcomes across different molecular subtypes [7, 8]. Thus, the preoperative identification of the breast cancer molecular subtype assumes paramount importance in facilitating early and specific treatment strategies, potentially prolonging survival and enhancing prognosis for young women with breast cancer.

Imaging examinations play a pivotal role in the comprehensive management of breast cancer, encompassing diagnosis, staging, treatment planning, and follow-up. Moreover, imaging modalities hold promise in predicting the molecular subtype of the tumor, thereby guiding treatment decisions. If the molecular subtype could be accurately determined through non-invasive imaging diagnostic methods, clinicians would be equipped to devise rational and individualized strategies, thereby optimizing treatment plans. The lower sensitivity of mammography in younger women, attributed to dense breast tissue, has propelled breast ultrasonography (US) as the preferred diagnostic method for women under 40 [9]. Shear wave elastography (SWE) represents an ultrasound quantitative imaging technology extensively employed in clinical oncology for the consistent measurement of tumor hardness [10]. Research suggests that elevated elastography parameters correlate with aggressive molecular subtypes [11, 12]. Although previous studies have explored the correlation between imaging features of breast cancer in young women and molecular subtypes [13, 14], there remains a paucity of research examining the relationship between ultrasonography combined with elastography and molecular subtypes in young women with breast cancer.

Thus, the primary objective of our study is to elucidate the correlation between ultrasonography combined with elastography parameters and the clinicopathological features and molecular subtypes of breast cancer in young women.

## 2. Materials and methods

### 2.1. Study participants

This retrospective study involved the analysis of routinely acquired ultrasound images, which received approval from the Shanxi Bethune Hospital Ethics Review Committee (No. SBQKL-2021-009). This study was conducted in accordance with the declaration of Helsinki. As the study involved the retrospective analysis of anonymized data, patient consent was deemed unnecessary by Shanxi Bethune Hospital Ethics Review Committee.

**Figure 1.**
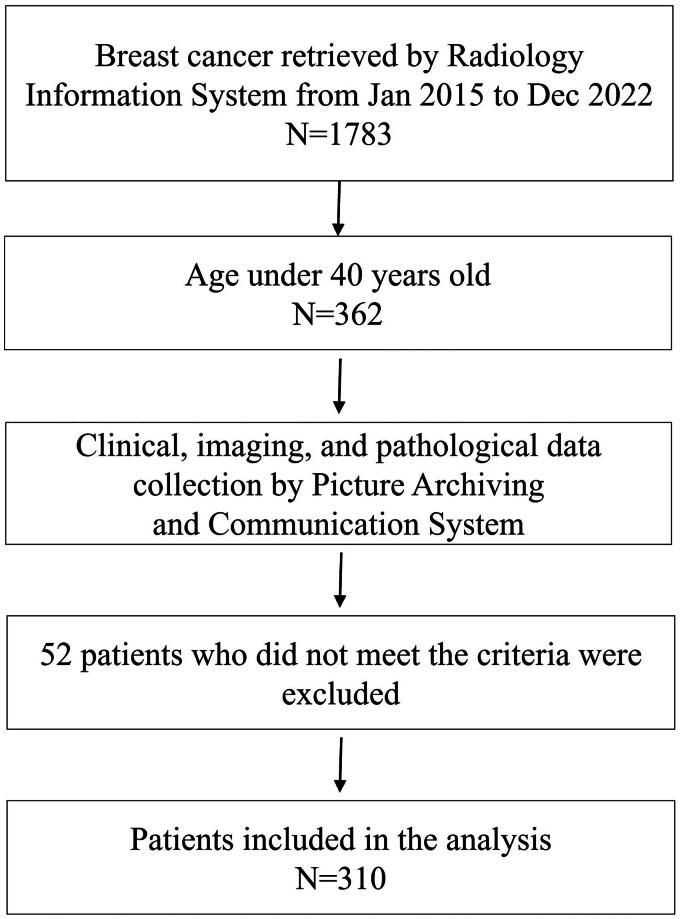
Flowchart of the study.

A total of 1783 female patients with breast cancer received treatment and follow-up at our hospital’s breast centre between January 2015 and December 2022, including 362 patients younger than 40 years old. According to the following criteria, 310 cases were enrolled in the study (Figure 1). Clinical, imaging and histopathological data were retrieved from the hospital information system for this study.

Inclusion criteria: (1) initial pathological diagnosis confirming primary breast cancer with a solitary unilateral breast lesion; (2) individuals who underwent surgical intervention with complete histopathological and immunohistochemical examination results; (3) patients with complete records of ultrasound imaging; (4) patients who received standardized treatment, including postoperative adjuvant chemotherapy, radiation treatment, endocrine therapy, targeted therapy, and consistent follow-up care.

Exclusion criteria: (1) patients with a secondary primary malignancy (*n* = 5); (2) patients who underwent neoadjuvant chemotherapy or endocrine therapy prior to surgery (*n* = 17); (3) patients unable to receive standardized treatment due to liver, kidney, or heart function abnormalities (*n* = 2); (4) patients with incomplete clinical or ultrasound examination data (*n* = 24); (5) patients with multiple lesions in a single breast (*n* = 4).

### 2.2. Ultrasound equipment and examination methods

The conventional US and SWE images were acquired using the Aixplorer system (SuperSonic Imagine, Aix en Provence, France) employing a 4–15 MHz linear transducer. Patients were positioned supine with their arms raised to fully expose both breasts and axillae. Bilateral breasts were meticulously examined using continuous cross-sectional, longitudinal section, and sector scanning techniques. Ultrasound image characteristics were meticulously observed and documented on the largest cross-section of the lesion, encompassing parameters of size, morphology, margins, posterior echo features, presence or absence of calcification, and blood flow grading. Lesions were classified in accordance with the fifth edition of BI-RADS (Breast-imaging-reporting and data system).

Following the acquisition of grayscale images SWE imaging of the breast lesions was performed. The built-in region of interest (ROI) in the system encompassed the lesion and surrounding normal tissue, generating a semi-transparent colour map of tissue stiffness superimposed on the grayscale image. In this colour map, deep blue denoted the lowest hardness while red indicated the highest. A fixed 4 × 4 mm ROI was placed on the hardest part of the lesion incorporating adjacent stiff tissue or halo and the system automatically computed the elasticity value of the lesion, measured in kilopascals (KPa), recording the maximum elasticity value (Emax). Each lesion underwent three measurements and the average value was subsequently calculated.

### 2.3. Image collection and analysis

Ultrasonography images were meticulously analyzed based on BI-RADS criteria, encompassing morphology, margins, posterior echo, blood flow grading, and the presence or absence of calcification. Morphological characteristics were categorized as regular and irregular, while margins were classified as smooth, lobulated, angular, and spiculated. The posterior echo features of lesions were classified as no change, enhancement, and attenuation. No change refers to the consistency of echoes with surrounding tissues at the same level. Enhancement refers to the increase in echo compared to surrounding tissues at the same level. Attenuation refers to a decrease in echo compared to surrounding tissues at the same level. Blood flow grading was stratified into four levels according to the Adler criteria [15]: grade 0 denoted no blood flow signal; grade 1 represented one to two short rod-like blood flow signals; grade 2 indicated three to four short rod-like blood flow signals; and grade 3 signified abundant blood flow, encompassing more than five signals or a large penetrating vessel. SWE image features were scrutinized to identify the hardest part of the lesion with the Emax value being measured and duly recorded. In general, the system’s built-in region of interest (ROI) includes lesions and surrounding normal tissues. When a few lesions were larger than the area of interest, the ROI was moved to find the reddest and brightest colour area for measurement. Measurements were taken three times for each lesion and the average value was calculated.

### 2.4. Histopathology and immunohistochemical analysis

The postoperative pathology reports of the enrolled patients were meticulously collected to procure their clinicopathological features, comprising histological type, cytological grading, lymph node metastasis, oestrogen receptor (ER) expression, progesterone receptor (PR) expression, human epidermal growth factor receptor 2 (HER2) expression, cellular proliferation index (Ki67) expression, and lymphovascular invasion. Tumor histological types were evaluated in accordance with the World Health Organization classification of breast tumors (fifth edition) [16], while histological grading was conducted utilizing the modified Bloom-Richardson grading system [17].

ER and PR positivity are defined using a cutoff of ≥ 1%. The intensity of HER2 expression is semi-quantitatively scored as 0, 1, 2, or 3. Tumors scoring 3 are categorized as HER2 positive, while those scoring 0 or 1 are classified as HER2 negative. Tumors scoring 2 necessitated gene amplification to ascertain HER2 status [18]. Ki67 positive expression is defined as Ki67 positivity in ≥ 14% of cancer cell nuclei.

Immunohistochemical molecular subtypes were determined based on the 2013 St. Gallen International Expert Consensus, classifying tumors into five subtypes: luminal A (ER+ and/or PR+, HER2−, and Ki67 < 14%); luminal B/HER2− (ER+ and/or PR+, HER2−, Ki-67 ≥ 14%); luminal B/HER2+ (ER+ and/or PR+, HER2+, any Ki67); HER2+ (ER-, PR-, HER2+); and triple-negative breast cancer (TNBC) (ER-, PR-, HER2−) [19].

The status of axillary lymph nodes was evaluated utilizing preoperative cytopathology, postoperative pathological results, and imaging examination. Histological diagnosis was conducted by a pathologist possessing over 10 years of expertise in breast histology, who remained uninformed regarding the clinicopathological features of the patient.

### 2.5. Statistical analysis

All data were subjected to statistical analysis using SPSS software (version 26.0; IBM Corp, Armonk, NY, USA). Continuous variables conforming to normal distribution are presented as mean ± standard deviation (±s), whereas those deviating from normality are expressed as medians (M) and interquartile ranges (P25, P75). Categorical variables are delineated as n (%). Analysis of variance (ANOVA) was employed when residuals between the continuous variables adhering to normal distribution, while the rank sum test was utilized for those not conforming to normality. Categorical variables were analyzed using the chi-squared (χ^2^) test. Comparisons across the five molecular subtypes were conducted for clinicopathological features and ultrasound imaging characteristics with a significance threshold set at *p* < 0.05.

Subsequently, binary logistic regression analysis was conducted, with 1 denoting the molecular subtype of interest and 0 representing the remaining four subtypes. Odds ratios (OR) and their corresponding 95% confidence intervals (CI) were calculated to identify ultrasound parameters associated with each molecular subtype.

## 3. Results

In a cohort of 310 young women diagnosed with breast cancer, each presented with a single lesion. The participants had an average age of 33.0 ± 3.42 years, ranging from 17 to 39 years, and the lesions had an average maximum diameter of 3.46 cm, with a range from 1.06 to 6.58 cm. The mean tumor hardness was recorded as 189.5 Kpa (range: 97.6–266.4 Kpa). Among the 310 cases, 29 (9.4%) were identified as pregnancy/lactation-associated breast cancers, and 273 (88.1%) presented with palpable tumors. Additionally, 14 patients underwent mutation testing for BRCA 1/BRCA 2 utilizing next-generation sequencing (NGS), with 10 testing positive and 4 testing negative.

### 3.1. Clinicopathological features of each molecular subtype

Among the 310 patients, 76.5% were diagnosed with tumors classified as T2 and T3 according to clinical TNM staging. The predominant histological type was invasive ductal carcinoma, accounting for 89.4% of cases, followed by ductal carcinoma *in situ* (4.8%), and invasive lobular carcinoma (2.9%). Notably, within the luminal A subtype, there were 2 cases of neuroendocrine cancer, while the luminal B (HER2−) subtype included 3 cases of solid papillary carcinoma and 4 cases of mucinous carcinoma. Grade 3 histological grading was the most prevalent, observed in 50.6% of cases. Axillary lymph node metastasis was detected in 48.7% of cases, while lymphovascular invasion was noted in 50.0% of cases.

The immunohistochemical molecular subtypes comprised 45 cases of luminal A subtype (14.5%), 90 cases of luminal B (HER2−) subtype (29.0%), 52 cases of luminal B (HER2+) subtype (16.8%), 57 cases of HER2+ subtype (18.4%), and 66 cases of TNBC (21.3%). Notably, the TNBC subtype exhibited the highest rates of axillary lymph node metastasis and lymphovascular invasion, at 69.7% and 60.6% respectively (*p* < 0.05). Specific data are delineated in Table 1.

**Table 1.** The clinicopathologic features of young breast cancer in five molecular subtypes (*n* = 310).

Parameter	Luminal A *n* = 45 (14.5)	Luminal B		HER2+ *n* = 57 (18.4)	TNBC *n* = 66 (21.3)	P
		HER2− *n* = 90 (29.0)	HER2+ *n* = 52 (16.8)			
Age	33.2 ± 2.83	33.1 ± 3.34	33.2 ± 4.17	33.1 ± 3.79	32.7 ± 2.97	0.920*
Histologic type						0.003^#^
IDC	41 (91.1)	72 (80.0)	47 (90.4)	54 (94.7)	63 (95.5)	
ILC	0	6 (6.7)	0	0	3 (4.5)	
DCIS	2 (4.4)	5 (5.6)	5 (9.6)	3 (5.3)	0	
Other	2 (4.4)	7(7.7)	0	0	0	
Clinical T stage n (%)						0.000^#^
I	22 (48.9)	17 (18.9)	9 (17.3)	17 (29.8)	8 (12.1)	
II	20 (44.4)	52 (57.8)	33 (63.5)	33 (57.9)	25 (37.9)	
III	3 (6.7)	23 (25.6)	10 (19.2)	7 (12.3)	33 (50.0)	
Histologic grade n (%)						0.000^#^
1	15 (33.3)	23 (25.6)	10 (19.2)	3 (5.3)	2 (3.0)	
2	30 (66.7)	36 (40.0)	15 (28.9)	9 (15.8)	10 (15.2)	
3	0	31 (34.4)	27 (51.9)	45 (78.9)	54 (81.8)	
Lymph node status n (%)						0.000^#^
Negative	29 (64.4)	55 (61.1)	35 (67.3)	20 (35.1)	20 (30.3)	
Positive	16 (35.6)	35 (38.9)	17 (32.7)	37 (64.9)	46 (69.7)	
Lymphovasular invasion n (%)						0.004^#^
Negative	33 (73.3)	48 (53.3)	25 (48.1)	23 (40.4)	26 (39.4)	
Positive	12 (26.7)	42 (46.7)	27 (51.9)	34 (59.6)	40 (60.6)	

Note: *ANOVA; ^#^ χ 2 test.

IDC, invasive ductal carcinoma; ILC, invasive lobular carcinoma; DCIS, intraductal carcinoma).

### 3.2. Ultrasound imaging features of each molecular subtype

Significant statistical disparities were evident in tumor size, morphology, margins, calcification, posterior echo features, blood flow (Adler grading), and tumor hardness among the five molecular subtypes (*p* < 0.05) (refer to Table 2).

**Table 2.** Ultrasonography features of young breast cancer in five molecular subtypes (*n* = 310).

US Parameter	Luminal A *n* = 45 (14.5)	Luminal B		HER2+ *n* = 57 (18.4)	TNBC *n* = 66 (21.3)	P
		HER2− *n* = 90 (29.0)	HER2+ *n* = 52 (16.8)			
Tumor size (cm, Mean ± SD)	2.87 ± 2.13	3.43 ± 2.28	3.36 ± 2.17	3.75 ± 2.45	3.84 ± 2.76	0.003*
Group of size n (%)						<0.001^#^
<2 cm	16 (35.6)	23 (25.6)	6 (11.5)	7 (12.3)	7 (10.6)	
2–5 cm	24 (53.3)	49 (54.4)	41 (78.9)	41 (71.9)	43 (65.2)	
>5 cm	5 (11.1)	18 (20.0)	5 (9.6)	9 (15.8)	16 (24.2)	
Shape n (%)						
Regular (oval/round)	4(8.9)	11 (12.2)	5 (9.6)	10 (17.5)	25 (37.9)	<0.001^#^
Irregular	41(91.1)	79 (87.8)	47 (90.4)	47 (82.5)	41 (62.1)	
Margin n (%)						<0.001^#^
Circumscribed	3 (6.7)	6 (6.7)	5 (9.6)	9 (15.8)	31 (47.0)	
Microlobulate	5 (11.1)	19 (21.1)	8 (15.4)	31 (54.4)	26 (39.4)	
Angular	13 (28.9)	48 (53.3)	30 (57.7)	14 (24.6)	9 (13.6)	
Spiculated	24 (53.3)	17 (18.9)	9 (17.3)	3 (5.2)	0	
Posterior features n (%)						<0.001^#^
No change	30 (66.7)	64 (71.1)	35(67.3)	35(61.4)	22(33.3)	
Enhance	6 (13.3)	11 (12.2)	7(13.5)	16(28.1)	44(66.7)	
Shandow	9 (20.0)	15 (16.7)	10(19.2)	6(11.5)	0(0)	
Calcification n (%)						<0.001^#^
Negative	37 (82.2)	48 (53.3)	25 (48.1)	23 (40.4)	42 (63.6)	
Positive	8 (17.8)	42 (46.7)	27 (51.9)	34 (59.6)	24 (36.4)	
Adler grade of blood flow n (%)						<0.001^#^
0–1	38 (84.4)	62 (68.9)	34 (65.4)	16 (28.1)	33 (54.5)	
2–3	7 (15.6)	28 (31.1)	18 (34.6)	41 (71.9)	30 (45.5)	
SWE max (Kpa)	165.92 ± 27.07	171.21 ± 30.03	183.64 ± 31.52	212.25 ± 28.37	204.76 ± 30.66	<0.001*

Note:*ANOVA; ^#^ χ 2 test.

Luminal A subtype tumors typically manifested as smaller lesions whereas TNBC tumors tended to be larger in size. Notably, over 60% of TNBC tumors exhibited a regular morphology, whereas other subtypes predominantly present with irregular morphologies. Smooth margins were frequently observed in TNBC tumors (approximately 47%), while luminal B subtype tumors frequently exhibited angular margins (exceeding 50%), luminal A subtype tumors exhibited spiculated margins (approximately 53.3%), whereas HER2+ subtype tumors tended to display lobulated margins (approximately 54%) (*p* < 0.001).

Furthermore, luminal subtypes and HER2+ subtype tumors predominantly demonstrate no change in posterior echo (over 60%), while enhanced posterior echo was more frequently observed in TNBC tumors (approximately 66.7%) (*p* < 0.001).

According to the Adler blood flow grading, the luminal A subtype exhibited lower blood flow grading, predominantly grades 0–1 (84.4%), whereas HER2+ and TNBC subtype predominantly exhibited grades 2–3. Notably, the HER2+ subtype demonstrated a richer blood flow compared to TNBC, with grades 2–3 accounting for 71.9% (*p* < 0.001).

Moreover, tumor hardness varied across different molecular subtypes, with the HER2+ subtype displaying the highest Emax value followed by TNBC, while luminal A and B subtypes exhibited lower Emax values (*p* < 0.001).

### 3.3. Binary logistic regression analysis for breast cancer specific molecular subtypes

**Figure 2.**
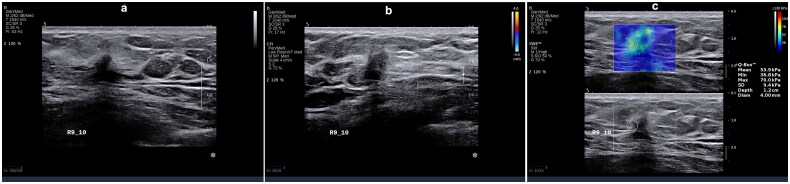
A 32-year-old female with right breast invasive ductal carcinoma grade II (score of 6), tumor size 1.0 × 0.8 × 0.5 cm, luminal a subtype. (a) Ultrasonography features: irregular shape, spiculated and angular margins, no change in posterior echo, no calcification. (b) Adler blood flow grading – grade 0 (c) Emax = 70.0 Kpa.

**Figure 3.**
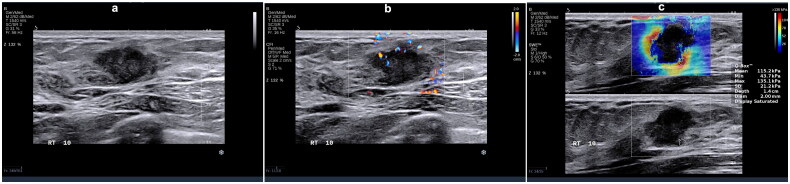
A 26-year-old female with right breast invasive ductal carcinoma grade III (score of 8), tumor size 2.0 × 1.5 × 1.0 cm, luminal B (HER2−) subtype. (a) Ultrasonography features: irregular shape, angular margins, no change in posterior echo, no calcification. (b) Adler blood flow grading – grade 1. (c) Emax = 135.1 Kpa.

**Figure 4.**
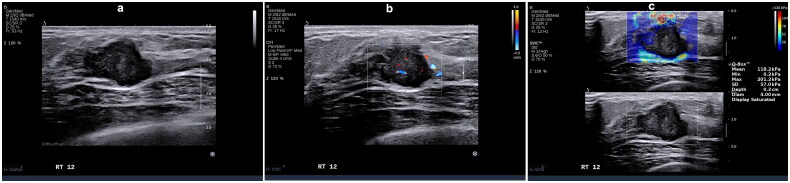
A 37-year-old female with right breast invasive ductal carcinoma grade II (score of 7), tumor size 2.0 × 1.8 × 1.5 cm, luminal B (HER2+) subtype. (a) Ultrasonography features: irregular shape, angular and lobulated margins, enhanced posterior echo, multiple punctate calcifications. (b) Adler blood flow grading – grade 2. (c) Emax = 201.2 Kpa.

The luminal A subtype exhibits a positive correlation with spiculated margins (*p* = 0.000, OR = 2.60) and a negative correlation with calcification (*p* = 0.003, OR = 0.25), blood flow grading (*p* = 0.039, OR = 0.37), and tumor hardness (*p* = 0.000, OR = 0.93). This implies that spiculated margins, lower blood flow grading, and lower hardness may serve as predictive indicators for the luminal A subtype (Figure 2). For the luminal B (HER2−) subtype there was positive correlation with angular margins (*p* = 0.002, OR = 1.60) and a negative correlation with tumor hardness (*p* = 0.000, OR = 0.97). Similarly, the luminal B (HER2+) subtype demonstrated a positive correlation solely with angular margins (*p* = 0.002, OR = 1.60). Therefore, regardless of HER2 status, angular margins may serve as a predictive factor for the luminal B subtype (Figures 3 and 4).

**Figure 5.**
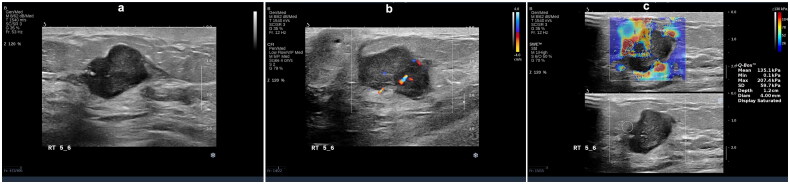
36-year-old female with left breast invasive ductal carcinoma grade III (score of 8), tumor size 2.2 × 1.6 × 1.3 cm, HER2+ subtype. (a) Ultrasonography features: irregular shape, lobulated margins, no change in posterior echo, multiple punctate calcifications. (b) Adler blood flow grading – grade 2. (c) Emax = 207.4 Kpa.

The HER2+ subtype displays positive correlations with calcification (*p* = 0.038, OR = 1.96), blood flow grading (*p* = 0.000, OR = 4.12), and tumor hardness (*p* = 0.000, OR = 1.02), indicating that tumors with higher blood flow grading, presence of calcification, and higher hardness are likely to be HER2+ subtype (Figure 5).

**Figure 6.**
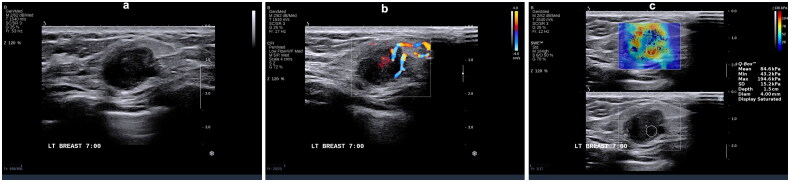
A 30-year-old female with left breast invasive ductal carcinoma grade III (score of 9), tumor size 1.7 × 1.5 × 1.5 cm, triple-negative breast cancer (TNBC). (a) Ultrasonography features: relatively regular morphology, relatively smooth margins, enhanced posterior echo, no calcification. (b) Adler blood flow grading – grade 3. (c) Emax = 194.6 Kpa.

Conversely, TNBC exhibits negative correlations with margins (*p* = 0.000, OR = 0.22), and calcification (*p* = 0.009, OR = 0.40), and a positive correlation with tumor hardness (*p* = 0.001, OR = 0.22). This suggests that tumors with smooth margins, no calcification, and higher hardness may be indicative of TNBC (Figure 6). Notably, tumor size, morphology, and posterior echo features show no significant correlation with any molecular subtype after logistic regression analysis (Table 3).

**Table 3.** Binary logistics regression analysis of molecular subtypes with ultrasound feature.

Molecular subtype	US parameter	b	P	OR	95%CI
Luminal A vs other subtype	Margin	0.96	0.000	2.60	1.58–4.29
	Calcification	−1.37	0.003	0.25	0.10–0.64
	Adler grade of blood flow	−1.01	0.039	0.37	0.14–0.95
	SWE max	−0.07	0.000	0.93	0.91–0.96
Luminal B HER2− vs other subtype	Margin	0.47	0.002	1.60	1.19–2.14
	SWE max	−0.03	0.000	0.97	0.96–0.99
Luminal B HER2+ vs other subtype	Margin	0.35	0.036	1.41	1.02–1.96
HER2+ vs other subtype	Calcification	0.67	0.038	1.96	1.04–3.70
	Adler grade of blood flow	1.42	0.000	4.12	2.13–7.98
	SWE max	0.02	0.000	1.02	1.01–1.03
TNBC vs other subtype	Margin	−1.52	0.000	0.22	0.14–0.33
	Calcification	−0.93	0.009	0.40	0.20–0.79
	SWE max	0.02	0.001	0.22	1.01–1.03

## 4. Discussion

Previous studies investigating breast cancer in young women have employed various age cutoffs, most commonly 35, 40, or 45 years [20, 21]. However, aligning with international consensus guidelines for young women with breast cancer, in this study we specifically targeted patients with breast cancer under the age of 40 [22]. It is noteworthy that compared to older patients with breast cancer, breast cancer in young women tends to exhibit higher histological grading. For instance, Bullier et al. [23] reported that the incidence rate of grade 2 and grade 3 breast cancer among young women is 94%, while Redmond et al. [24] reported an incidence rate of 87%, findings that are consistent with our observed rate of 82.9%.

In our study, the distribution of molecular subtypes among young women with breast cancer was as follows: luminal A subtype accounted for 14.5%, luminal B subtype for 45.8%, HER2+ subtype for 18.4%, and TNBC for 21.3%. Notably, the luminal B subtype was the most prevalent and the incidence rate of TNBC exceeded that of HER2+ subtype, which is consistent with the findings reported in previous studies [25, 26]. However, some studies suggest a higher prevalence of luminal A subtype among younger patients, potentially reaching up to 60%, alongside a higher incidence of HER2+ subtype and a lower incidence of TNBC [13, 23]. These discrepancies may stem from variations in the age ranges selected for inclusion in specific studies and the inherent heterogeneity of the disease.

Breast cancer in young women often exhibits with more aggressive histopathological features and poorer prognoses. Among the molecular subtypes, the luminal A subtype typically exhibits the highest survival rates, followed by the luminal B subtype, whereas the HER2+ subtype and TNBC tend to have lower survival rates [27]. The poor prognoses are often attributed to late-stage diagnoses and the presence of axillary lymph node or distant organ metastases. In our cohort, the overall rates of axillary lymph node metastasis and lymphovascular invasion were 48.7% and 50%, respectively, with TNBC demonstrating the highest rates among the subtypes at 69.7% and 60.6%, respectively. Hence, early detection of breast cancer in young individuals and the provision of individualized precision treatment based on different molecular subtypes are imperative for improving prognosis.

Currently, research on the relationship between molecular subtypes of breast cancer in young women and ultrasonographic imaging features is limited [13, 23, 24]. In our study, spiculated margins were frequently observed in the luminal A subtype (approximately 53.3%), while angular margins were more prevalent in the luminal B subtype (exceeding 50%). Logistic regression analysis revealed a positive correlation, consistent with findings reported in prior studies [28, 29]. Spiculated margins arise from the infiltration of cancerous tissue into surrounding areas leading to stromal reaction and fibrous connective tissue hyperplasia. This serves as an early protective mechanism that can somewhat limit the spread of cancer cells [30]. However, due to the scarcity of related studies the current conclusions necessitate further exploration and evaluation.

Previous studies have suggested that TNBC can exhibit characteristics resembling benign lesions, such as smooth or slightly lobulated margins and enhanced posterior echo [31, 32]. In this study, a majority of TNBC cases exhibited smooth margins (approximately 47%) and commonly exhibited enhanced posterior echo (approximately 66.7%). Notably, blood flow of grades 2 and 3 accounted for 45.5%, which was lower than that observed in the HER2+ subtype (63.2%), consistent with previous findings. The reduced blood flow distribution in TNBC compared to the HER2+ subtype may be attributed to higher cell density and necrosis [14].

The HER2+ subtype predominantly exhibited lobulated margins (approximately 54%), exhibited richer blood flow with grades 2 and 3 accounting for 63.2%, and presented with calcifications in approximately 59.6% of cases. Logistic regression analysis indicated a positive correlation between blood flow grading and calcification with the HER2+ subtype. Studies have suggested that HER2 overexpression is closely associated with increased angiogenesis and is linked to the expression of vascular endothelial growth factor (VEGF), which is also closely associated with calcifications, consistent with findings in this study [7, 33, 34]. However, Temel et al. [35] proposed that the HER2+ subtype displays less blood flow distribution compared to other subtypes, emphasizing the need for further large-scale studies to validate these observations.

Regarding the utilization of elastography to differentiate between molecular subtypes, existing studies have yet to produce conclusive results for accurately distinguishing among molecular subtypes. Chang et al. observed that breast cancers with aggressive prognostic features tend to exhibit higher hardness compared to those with favourable prognostic characteristics [11]. Zheng et al. suggested that aggressive molecular subtypes are associated with higher mean elasticity values (Emean) [36]. Additionally, Liu et al. noted that HER2− positive tumors tend to have higher Emean values compared to other subtypes [37]. Yoo et al. proposed that tumor hypoxia could potentially explain the increased tumor hardness observed in TNBC or HER2+ subtype tumors, demonstrating higher hardness than luminal subtypes [38].

In this study, we observed that the HER2+ subtype displayed the highest Emax value, followed by TNBC, while luminal A and B subtypes exhibited lower Emax values. Logistic regression analysis indicated that luminal A and luminal B (HER2−) subtypes are negatively correlated with tumor hardness, whereas HER2+ and TNBC subtypes were positively correlated with tumor hardness. However, Ganau et al. argued that lesion hardness is significantly associated with its size, with larger lesions exhibiting higher Emax values and that the hardness of TNBC and HER2+ subtypes is notably lower than that of luminal subtypes, although this finding was not statistically significant [39]. Kurt et al. found that compared to luminal A and B subtypes, HER2+ and TNBC tend to have relatively higher average Emax values, although no significant statistical difference was observed among all subtypes [40].

Due to variations in patient characteristics, selected ultrasound parameters, and discrepancies among study findings the conclusions remain inconclusive. Hence, further large-scale multicentre studies are warranted to validate these findings.

## 5. Limitations

Primarily, this investigation constitutes a single-centre retrospective study potentially introducing sample selection bias. Consequently, the prospective validation of ultrasound parameters for predicting the molecular subtypes of breast cancer in young women mandates comprehensive multicentre studies. Furthermore, ensuring the reproducibility and standardization of ultrasound parameters necessitates further exhaustive research for confirmation.

Secondarily, this investigation exclusively utilized ultrasound imaging technology. In the future, combining multiple imaging techniques such as X-rays and MRI for research should provide a more comprehensive understanding of the imaging characteristics of different molecular subtypes, and the findings will be more reliable and generalizable.

## 6. Conclusion

Distinct ultrasonography features, in conjunction with elastography parameters, exhibit correlations with specific breast cancer molecular subtypes. The integration of ultrasound examinations holds promise in non-invasively predicting the biological characteristics and prognosis of tumors thereby furnishing an imaging foundation for the early diagnosis and personalized treatment of breast cancer in young women.

## Data Availability

The datasets used and/or analyzed during the current study are available from the corresponding author upon reasonable request.

## Abbreviations

US: ultrasonography
SWE: shear wave elastography
HIS: hospital information system
BI-RADS: breast-imaging-reporting and data system
ROI: region of interest
ER: oestrogen receptor
PR: progesterone receptor
HER2: human epidermal growth factor receptor 2
Ki67: cellular proliferation index
TNBC: triple negative breast cancer
VEGF: vascular endothelial growth factor
